# Melatonin enhances resistance to *Botryosphaeria dothidea* in pear by promoting jasmonic acid and phlorizin biosynthesis

**DOI:** 10.1186/s12870-024-05187-1

**Published:** 2024-05-29

**Authors:** Hongpeng Xu, Siying Zhang, Chenglin Liang, Min Li, Ran Wang, Jiankun Song, Zhenhua Cui, Yingjie Yang, Jianlong Liu, Dingli Li

**Affiliations:** 1 Qingdao Key Laboratory of Genetic Improvement and Breeding in Horticultural Plants, Engineering Laboratory of Genetic Improvement of Horticultural Crops of Shandong Province, College of Horticulture, Qingdao Agricultural University, Qingdao, 266109 China; 2 Haidu College, Qingdao Agricultural University, Laiyang, 265200 China

**Keywords:** Melatonin, Pear, *Botryosphaeria dothidea*, Jasmonic acid, Phlorizin, Disease resistance

## Abstract

Ring rot, caused by *Botryosphaeria dothidea*, is an important fungal disease of pear fruit during postharvest storage. Melatonin, as a plant growth regulator, plays an important role in enhancing the stress resistance of pear fruits. It enhances the resistance of pear fruits to ring rot by enhancing their antioxidant capacity. However, the underlying mechanism remains unclear. In this study, we examined the effect of melatonin on the growth of *B. dothidea*. Results showed that melatonin did not limit the growth of *B. dothidea* during in vitro culture. However, metabolomics and transcriptomics analyses of ‘Whangkeumbae’ pear (*Pyrus pyrifolia*) revealed that melatonin increased the activity of antioxidant enzymes, including peroxidase (POD), superoxide dismutase (SOD), and polyphenol oxidase (PPO), in the fruit and activated the phenylpropanoid metabolic pathway to improve fruit resistance. Furthermore, melatonin treatment significantly increased the contents of jasmonic acid and phlorizin in pear fruit, both of which could improve disease resistance. Jasmonic acid regulates melatonin synthesis and can also promote phlorizin synthesis, ultimately improving the resistance of pear fruit to ring rot. In summary, the interaction between melatonin and jasmonic acid and phlorizin enhances the antioxidant defense response and phenylpropanoid metabolism pathway of pear fruit, thereby enhancing the resistance of pear fruit to ring rot disease. Our results provide new insights into the application of melatonin in the resistance to pear fruit ring rot.

**Supplementary Information:**

The online version contains supplementary material available at 10.1186/s12870-024-05187-1.

## Introduction

Pear is an important economic tree species in the world [1]. ‘Whangkeumbae’ pear (*Pyrus pyrifolia*) is widely cultivated in China, because of its excellent quality. In recent years, it has been found that pears were severely infected by *Botryosphaeria dothidea* during postharvest storage. *B. dothidea* causes branch withering, stem ulcers, and fruit decay, greatly affecting the commercial value of ‘Whangkeumbae’ pear fruit [2–4].

Biological measures are important for controlling fruit fungal diseases, among which postharvest resistance induction is an important method [5, 6]. Plant induced resistance, also known as induced systemic resistance, is a fast and strong defense response of plants against pathogen [7–9]. Following treatment with biological or chemical elicitors, plants gain the ability to express defense responses faster and stronger, enabling them to cope with biotic and abiotic stresses. This has been proven to be a common feature of the plant immune system that confers plants the ability to self-protect from bacterial pathogens, insects, and abiotic stresses, and also serves as an important cellular mechanism for plant-induced disease resistance. Plant defense response elicitors such as jasmonic acid (JA) and its functional analogs can keep plants in a sensitive state, triggering faster and stronger defense responses upon pathogen infection [10, 11]. Melatonin, as a novel plant growth regulator, has been reported to enhance biological stress resistance in various crops in recent years. For example, exogenous melatonin application inhibited mycelial growth and conidial development of powdery mildew on the fruit of watermelon and fruit and leaves of zucchini [12, 13]. Melatonin can significantly inhibit the occurrence of apple fruit damage caused by *P. expansum* inoculation [14]; melatonin could eliminate excess reactive oxygen species (ROS) produced in plants after pathogen infection, form an antioxidant cascade reaction, and alleviate oxidative damage caused by external stress [15–17]; and melatonin could enhance the antioxidant level in pear fruit following *B. dothidea* infection, further enhancing resistance [18]. Strawberries treated with melatonin increased the activity of antioxidant enzymes (APX, SOD, and POD) and decreased infection of *B. cinerea* [19].

Melatonin can induce the acquisition of systemic resistance in plants through the synthesis of resistance elicitors such as salicylic acid (SA), further activating plant defense responses. Melatonin can also induce systemic resistance through JA to enhance plant immunity. For example, melatonin can induce JA synthesis during the infection process of the model plant *Arabidopsis thaliana* [20]. Melatonin increased the accumulation of JA in bananas, inducing resistance to banana wilt disease [21]. In tomato, melatonin treatment increased methyl jasmonate (MeJA) content and upregulated the expression of *SlLoxD*, *SlAOC*, and *SlPI II* genes to attenuate fruit fungal decay [22]. Melatonin treatment significantly inhibited gray mold development and induced disease resistance in cherry tomato fruit during storage by enhancing SA biosynthetic enzyme activity and consequently SA accumulation [23].

In addition, melatonin plays an important role in regulating the synthesis of secondary metabolites in plants [24, 25]. For instance, melatonin promotes the content of flavonoids, total phenols, and lignin in tomatoes, regulates the metabolic pathway of phenylpropanoic acid, and increases the resistance of tomatoes to pathogenic fungi such as *Botrytis* after harvest [26]. Melatonin enhances the resistance of cotton to *Verticillium dahliae*, which is related to the biosynthesis of lignin and gossypol in cotton [27]. Melatonin promotes the accumulation of flavonoids, total phenols, lignin, and anthocyanins in blueberry fruits, thereby increasing their post harvest disease resistance [28]. These studies indicate that melatonin induces disease resistance in plants by affecting the signal transduction of plant metabolites. However, a systematic exploration of the effect of melatonin on fruit disease resistance at the metabolic and transcriptional levels is lacking.

This study investigated the effect of exogenous melatonin treatment on the resistance of pear fruit to *B. dothidea*. The effect of melatonin on fungal growth was determined through in vitro fungal culture. Additionally, through systematic metabolomics and transcriptomics analyses, we explored the role of melatonin in improving the resistance of pear fruit to *B. dothidea* and validated the relationship between its effects.

## Materials and methods

### Plant material and treatment methods

Commercially mature ‘Whangkeumbae’ pear (*Pyrus pyrifolia*) fruits of moderate size and with no obvious damage were used in this study. This study used commercially matured pear fruits, which were of moderate size without significant damage, and had a flesh hardness(kg/cm^2^) of 8.49 ± 0.72; SSC (%) is 9.2 ± 0.5, and starch content (mg/g) is 3.62 ± 0.5. The selected fruits are disinfected with 2% sodium hypochlorite for 1 min on the surface, rinsed twice with sterile water for inoculation, and then wiped dry. Six soaking treatments were performed, each with more than 20 fruits. The fruits were soaked at 25℃ for 12 h in distilled water (control), 100 µmol/L melatonin (MT), 100 µmol/L MeJA (hereafter denoted as JA treatment), 20 mmol/L phlorizin, 150 µmol/L DL-4-chlorophenylalanine (PCPA, melatonin inhibitor), or 100 µmol/L salicylhydroxamic acid (DIECA, JA inhibitor). After soaking, use a sterile punch with a diameter of 4.75 mm to punch holes in the center area of the pear fruit, and stick fungal onto the punched area (day 1). Fruits were incubated at 26.5℃ and 85% relative humidity for observation, and samples were collected at 1, 3, and 5 d. The samples were immediately frozen in liquid nitrogen and stored at − 80℃ for subsequent analyses.

### Observation of *B. dothidea* mycelia

The fungal pathogen *B. dothidea* was grown on potato dextrose agar (PDA) medium containing sterile water (CK) or different concentrations of melatonin (50, 100, and 200 µmol/L) or phlorizin (0.02, 0.2, 2, and 20 mmol/L). The cultures were observed, and photographs were taken on 1, 3, and 5 day. To perform scanning electron microscopy, *B. dothidea* mycelia cultured for 5 days were fixed in 2.5% glutaraldehyde and dehydrated in an ethanol gradient (50%, 70%, 90%, and 100%). After dehydration, ethanol was replaced with 100% tert butanol. Subsequently, a 3 mm × 6 mm piece of *B. dothidea* mycelium was placed on a glass slide for 24 h and then coated with gold/palladium. The morphology of mycelium was observed using a scanning electron microscope (JSM-7500 F, Japan Electronics, China).

### Observation of *B. dothidea* growth in pear fruits

On the 5 day, the flesh of *B. dothidea*-inoculated pear fruits was sampled at the junction of normal and infected parts and fixed in formalin-acetic acid-alcohol fixative (FAA). The fixed fruit tissue was rinsed with distilled water, and then observed using the EVOS™ FL Auto 2 Imaging System™ (EVOS FL AUTO 2.0, Thermo, USA).

### Quantification of total melatonin and JA

The content of melatonin and jasmonic acid was determined using the double antibody sandwich method. Extract fruit pulp (0.25 g) using 2.25 mL phosphate buffer (pH 7.4). Purified plant jasmonic acid (JA) or melatonin (MT) antibodies are coated on a microplate to prepare solid-phase antibodies. Jasmonic acid (JA) or melatonin (MT) is sequentially added to the microplate of the coated monoclonal antibody, and then combined with Horseradish Peroxidase labeled jasmonic acid (JA) or melatonin (MT) antibodies. After thorough washing, substrate is added for color development. Measure the absorbance (OD value) at a wavelength of 450 nm using an enzyme-linked immunosorbent assay kit (Tongwei Biotechnology, Shanghai, China) to detect endogenous melatonin and JA.

### Detection of antioxidant metabolites

Weigh fresh pear meat sample (0.2 g) and homogenize it with liquid nitrogen. Centrifuge the crude extract (1.8 mL) at 4000 g for 30 min at 4℃. Transfer the supernatant to a 2 mL centrifuge tube and place it on ice. Use the following method for measurement: The determination of POD content adopts the principle of peroxidase (POD) catalyzed hydrogen peroxide reaction, and colorimetric measurement is carried out at a wavelength of 420 nm through colorimetric analysis. Using the POD assay kit (JianCheng A084-3-1, Nanjing, China) to determine the POD content.

The determination of PPO content adopts the principle of polyphenol oxidase (PPO) catalyzing the production of quinone from substrate phenols, and colorimetric measurement is carried out at a wavelength of 420 nm through colorimetry. Using the PPO assay kit (JianCheng A136-1-1, Nanjing, China) to determine the PPO content.

The determination of CAT content adopts the principle of catalase (CAT) catalyzing the production of water and oxygen from hydrogen peroxide, and colorimetric measurement is carried out at a wavelength of 510 nm through colorimetry. Use the CAT assay kit (Grace Biotechnology G0105F, Jiangsu, China) to determine the CAT content.

### RNA extraction and transcriptome sequencing

Total RNA was extracted from samples using the Plant Total RNA Isolation Kit (TaKaRa, Beijing, China), according to the manufacturer’s instructions, and three biological replicates were performed for each sample. The quality of total RNA was verified using the 2100 Bioanalyzer system (Agilent, USA), and 1 mg of each sample was used for cDNA library construction. Library quality was assessed with the 2100 Bioanalyzer system (Agilent, Santa Clara, CA, USA). A clean dataset was obtained by removing reads containing adapter sequences, poly-Ns, and low-quality reads from the raw data. The Q20 and Q30 scores and GC content of the clean data were calculated. All downstream analyses were performed using the high-quality clean data. Reference genome and gene model annotation files were downloaded from the genome website (https://www.rosaceae.org/species/pyrus_bretschneideri/genome_v1. 1). An index of the reference genome was generated using HISAT2 v2.0.5, and paired-end clean reads were aligned to the reference genome. Feature Counts v1.5.0-p3 was used to count the number of reads mapped to each gene. The fragments per kilobase of exon per million mapped fragments (FPKM) value of each gene was calculated based on the length of the gene and number of reads mapped to the gene. Differential expression analysis of genes between two conditions/groups (three biological replicates per condition) was performed using the DESeq2 R package (v1.20.0). Gene ontology (GO) enrichment analysis of differentially expressed genes (DEGs) was implemented with the clusterProfiler R package, after correcting for the gene length bias. Statistical enrichment of DEGs in KEGG pathways was detected with the clusterProfiler R package.

### Metabolite extraction and profiling analysis

Control and *B. dothidea*-inoculated pears treated with melatonin were subjected to targeted metabolomic analysis. Place a total of 12 ± 0.2 g of stored samples in a vacuum freeze-drying machine (Scientz-100 F) for vacuum freeze-drying. Grind (30 Hz, 1.5 min) using a grinder (MM400, Retsch) to a powder state. Weigh 100 mg of powder and dissolve it in 1.2 mL of 70% methanol extraction solution. To ensure complete suspension of the sample, vortex the sample every 30 min for 30 s for a total of 6 times. The sample was refrigerated overnight at 4℃ and centrifuged at 12,000 rpm for 10 min. Suck the supernatant and use a microporous filter membrane (0.22 μm) Filter the sample and store it in the injection bottle. The resulting filtrate is used for the measurement of widely targeted metabolites. In metabolomics, the ultra-high performance liquid chromatography(SHI-MAZU Nexera X2;https://www.shimadzu.com.cn/) and tandem mass spectrometry methods and AppliedApplied Biosystems 4500 QTRAPhttp://www.appliedbiosystems.cn/) were performed by Wuhan Metware Biotechnology Co., Ltd. (Metware, Wuhan, China). To ensure robustness, three biological replicates were performed on each sample. Identify differential cumulative metabolites based on the following three parameters: log_2_ (multiple change) ≥ 1; Variable Importance Map (VIP) ≥ 1.0; Mann Whitney test *P* ≤ 0.05. KEGG analysis is used to understand the metabolic pathways after melatonin treatment.

### Real-time quantitative PCR (RT-qPCR) analysis

Total RNA was extracted from pear samples using the RNAprep Pure Plant Kit (Tiangen Biotech Co., Beijing, China), and cDNA was synthesized using the HiScript II 1st Strand cDNA Synthesis Kit (Vazyme Biotech Co., Nanjing, China). Then, RT-qPCR was performed on the Lightcycler® 480 II System (Roche, Basel, Switzerland) using the ChamQ SYBR Color qPCR Master Kit (Vazyme Biotech Co.) in a 20 µL reaction volume containing 2 µL of cDNA (template), 1 µL each of forward and reverse primers, 10 µL of Supermix, and 6 µL of RNA-free water. The reaction protocol was as follows: 95 °C for 5 min, then 45 cycles at 95 °C for 15 s, 60 °C for 30 s, and 72 °C for 30 s. The *Actin* gene was used as an internal control. The relative expression level of each gene was calculated with the 2^−ΔΔCT^ method. Each sample analysis was repeated three times. The primers used are listed in supplementary Table S1.

### Statistical analysis

Column charts were constructed using GraphPad Prism 6.01. Data were subjected to analysis of variance (ANOVA), and mean values were compared by Duncan’s test (*P* < 0.05). The SPSS 23.0 statistical software package (IBM SPSS Statistics, Chicago, IL, USA) was used for statistical analyses. All experimental data were obtained from three or more biological replicates.

## Results

### Effect of melatonin on the growth of *B. dothidea* mycelium

**Fig. 1:**
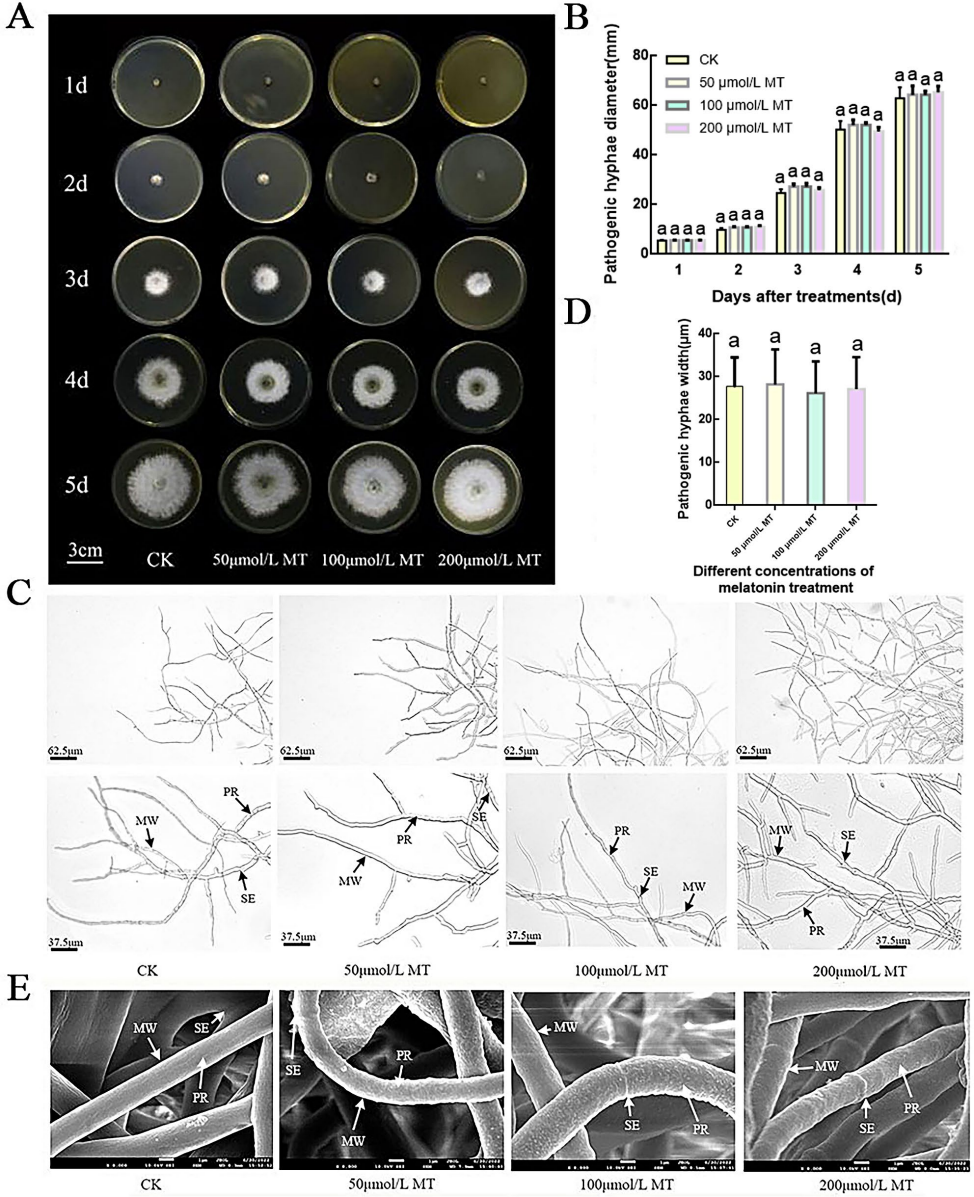
Melatonin has no significant effect on the growth of *B. dothidea* mycelium. (**A**) Growth of *B. dothidea* on PDA medium containing different concentrations of melatonin. (**B**) *B. dothidea* lesion diameter under different concentrations of melatonin. (**C**, **D**) Microscopic observation of *B. dothidea* mycelial structure (**C**) and thickness (**D**). (**E**) Transmission electron microscopy observation of *B. dothidea* mycelial structure. Different letters indicate significant differences, according to one-way ANOVA Duncan’s multiple range test (*P* < 0.05)

*B. dothidea* showed normal growth on PDA medium containing different concentrations of melatonin, and no significant difference was observed in *B. dothidea* plaque diameter within 5 days (Fig. 1A, B). Microscopic analysis revealed that the structure of melatonin-treated *B. dothidea* mycelium was intact, with clearly distinguishable membrane, protoplasm, and mycelium wall. The mycelium plumpness was consistent, and growth was normal (Fig. 1C, D). Scanning electron microscopy showed that the mycelium of *B. dothidea* in different treatments had a tubular structure, with a certain degree of curvature (Fig. 1E). This indicates that melatonin does not affect the growth of *B. dothidea*.

### Effect of melatonin on the resistance of pear fruit to *B. dothidea*

**Fig. 2:**
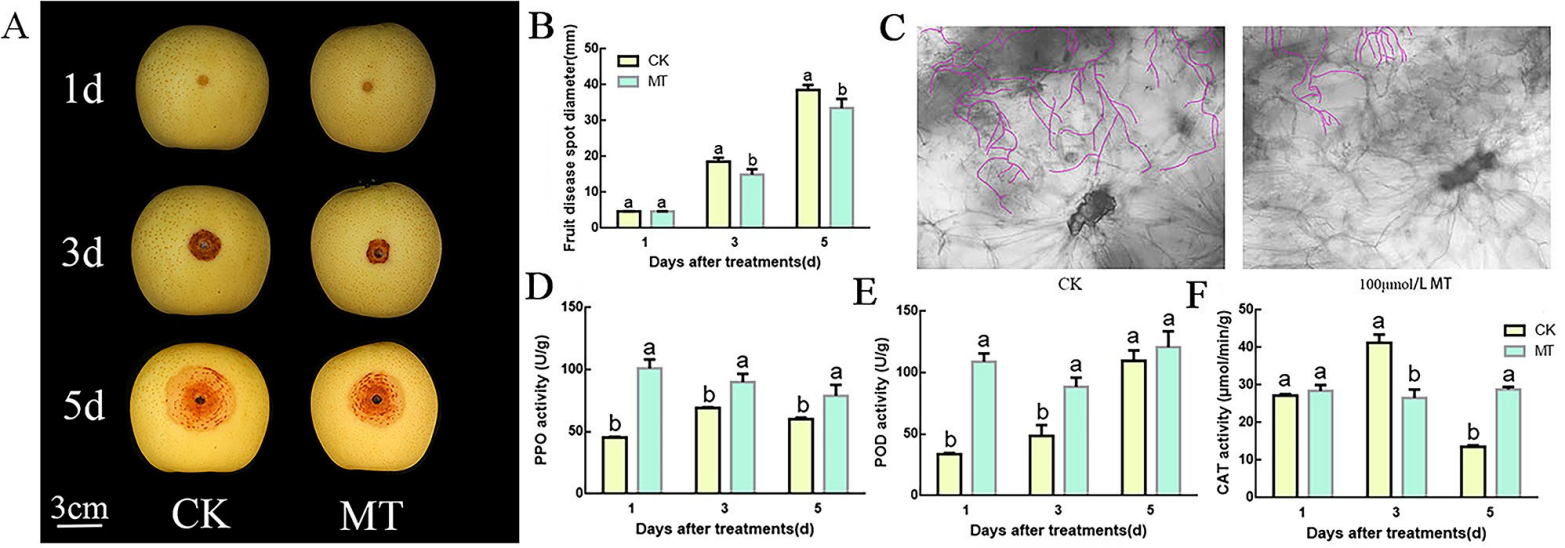
Melatonin inhibits mycelial growth in ‘Whangkeumbae’ fruits and enhances antioxidant capacity. (**A**) Growth of *B. dothidea* disease spots on the surface of ‘Whangkeumbae’ fruit after melatonin treatment. (**B**) Diameter of disease lesions on the surface of melatonin-treated fruit. (**C**) Spread of *B. dothidea* hyphae in ‘Whangkeumbae’ fruit pulp in the melatonin treatment. (**D**–**F**) Activity of antioxidant enzymes including PPO (**D**) POD (**E**), and CAT (**F**) in control and melatonin-treated fruit. Different letters indicate significant differences, according to one-way ANOVA Duncan’s multiple range tests (*P* < 0.05)

After *B. dothidea* infects the fruit, the diameter of disease spots on the surface of the fruit continues to increase. Compared with the control, melatonin treatment significantly reduced the diameter of lesions on the pear fruit surface (Fig. 2A, B). Melatonin treatment significantly reduced the density of hypha in the area between the infected and normal fruit flesh.

The activity of antioxidant enzymes including POD and PPO was significantly higher in melatonin-treated fruit than in the control fruit on day 1, and that of PPO was significantly higher in melatonin-treated fruit on day 5. POD activity in the control fruit increased to the level observed in the melatonin treatment group on the fifth day. No significant difference in CAT activity was observed after melatonin treatment until the fifth day, when the treatment group showed significantly higher activity than the control group (Fig. 2C and E).

### Effects of melatonin on *B. dothidea*-infected ‘Whangkeumbae’ fruit at the metabolic and transcriptional levels

**Fig. 3:**
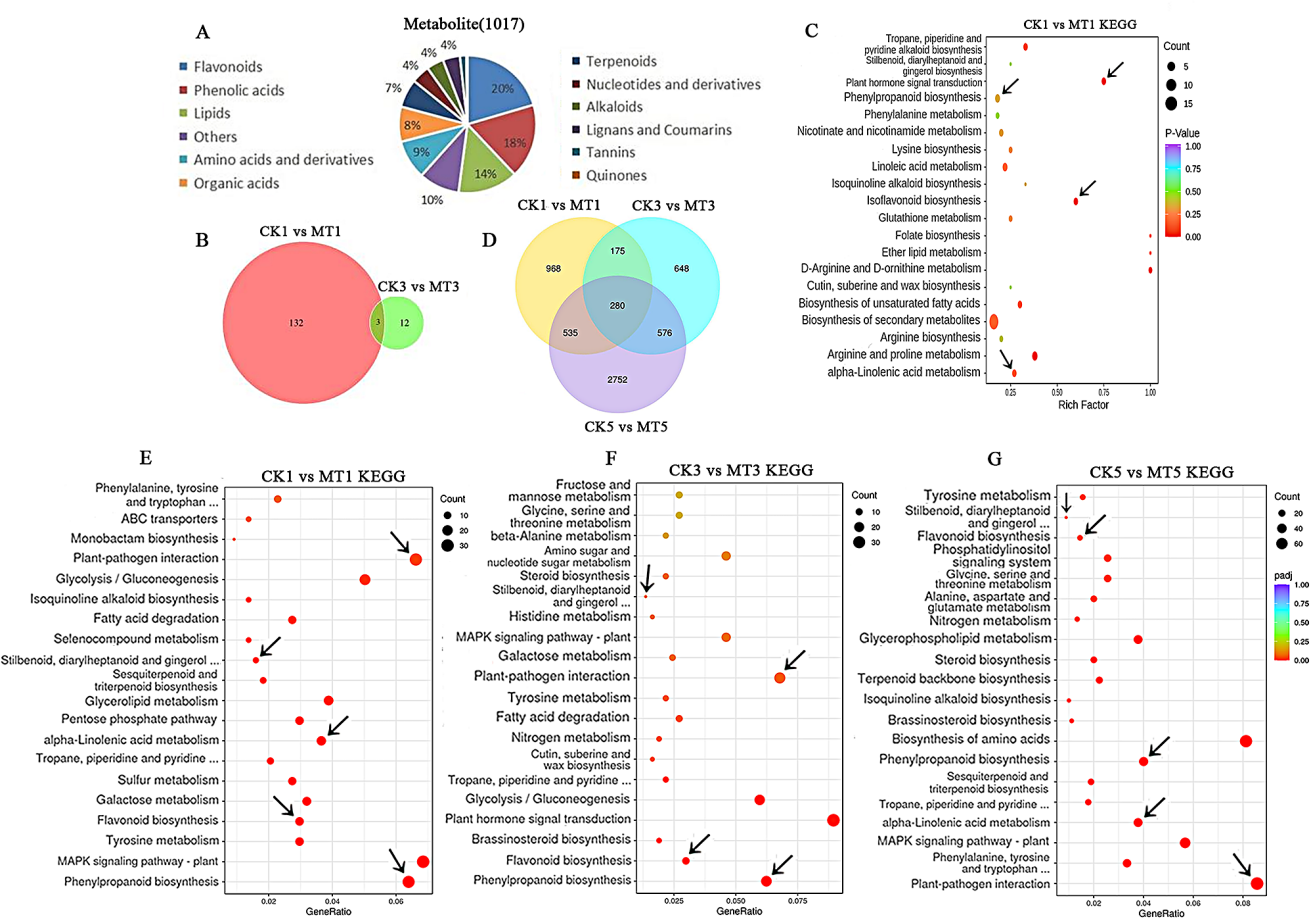
Analysis of metabolomics and transcriptomics data. (**A**) Distribution of total metabolites in pear fruits. (**B**) Venn diagram showing the number of DAMs identified in the CK1vsMT1 and CK3vsMT3 comparisons. (**C**) KEGG enrichment analysis of DAMs identified in the CK1vsMT1 comparison. Count refers to the number of DEGs annotated according to the KEGG database. (**D**) Venn diagram showing the number of DEGs identified in the MT1vsCK1, MT3vsCK3, and MT5vsCK5 comparisons. (**E**–**G**) KEGG pathway enrichment analysis of DEGs identified in the MT1vsCK1, MT3vsCK3, and MT5vsCK5 comparisons. Arrows indicate pathways related to disease resistance

Based on the effect of melatonin on the antioxidant capacity of pear fruit on the first and third days after fungal infection, we measured the metabolic changes in the fruit on days 1 and 3. Metabolomics analysis showed that a total of 1,017 metabolites were detected (Fig. 3A). The results of principal component analysis (PCA) showed that the first principal component PC1 could explain 37.59% of the total variance. At the same time, the PC1 value of the quality control sample is within the range of plus or minus 3 standard deviations, indicating that the instrument is in a stable state. A repeated correlation evaluation was conducted on the sample data, and it was found that the correlation coefficients of each group of repeated samples were high, indicating the reliability of the obtained differential metabolites (Figure S2). Therefore, further data analysis was conducted on metabolites. Based on 0.5 ≤ FC ≥ 2 and VIP ≥ 1, 135 metabolites were identified as DAMs between CK1 and MT1 (83 upregulated and 52 downregulated), and 15 metabolites were identified as DAMs between CK3 and MT3, (5 upregulated and 10 downregulated) (Fig. 3B). This indicates a significant change in metabolism within the fruit on day 1, and a decrease in the number of DAMs on day 3. Therefore, KEGG annotation analysis was conducted on the DAMs identified between the control and melatonin treated groups on day 1. The results showed that the DAMs in the CK1vsMT1 comparison were significantly enriched in the ‘biosynthesis of phenylpropane’, ‘α- Metabolism of linolenic acid’, and ‘flavonoid biosynthesis’ pathways (Fig. 3C).

Next, we conducted transcriptome analysis of fruits at different stages. Transcriptome data analysis revealed 968, 648, and 2,752 DEGs in the MT1vsCK1, MT3vsCK3 groups, and MT5vsCK5 comparisons (Fig. 3D). KEGG enrichment analysis showed that after melatonin treatment, these DEGs were significantly enriched in flavonoid biosynthesis, phenylpropane biosynthesis, α-linolenic acid metabolism, and plant-pathogen interaction biosynthesis pathways. On days 3 and 5, the differences in disease resistant metabolic pathways [29, 30] between the control and melatonin treatment groups decreased, indicating that melatonin treatment induced the expression of multiple genes involved in plant defense and secondary metabolism in pear fruit at the early stage (Fig. 3E and G).

### Joint metabolomic and transcriptomic analysis of key disease resistance related pathways in pear fruits after melatonin treatment

**Fig. 4:**
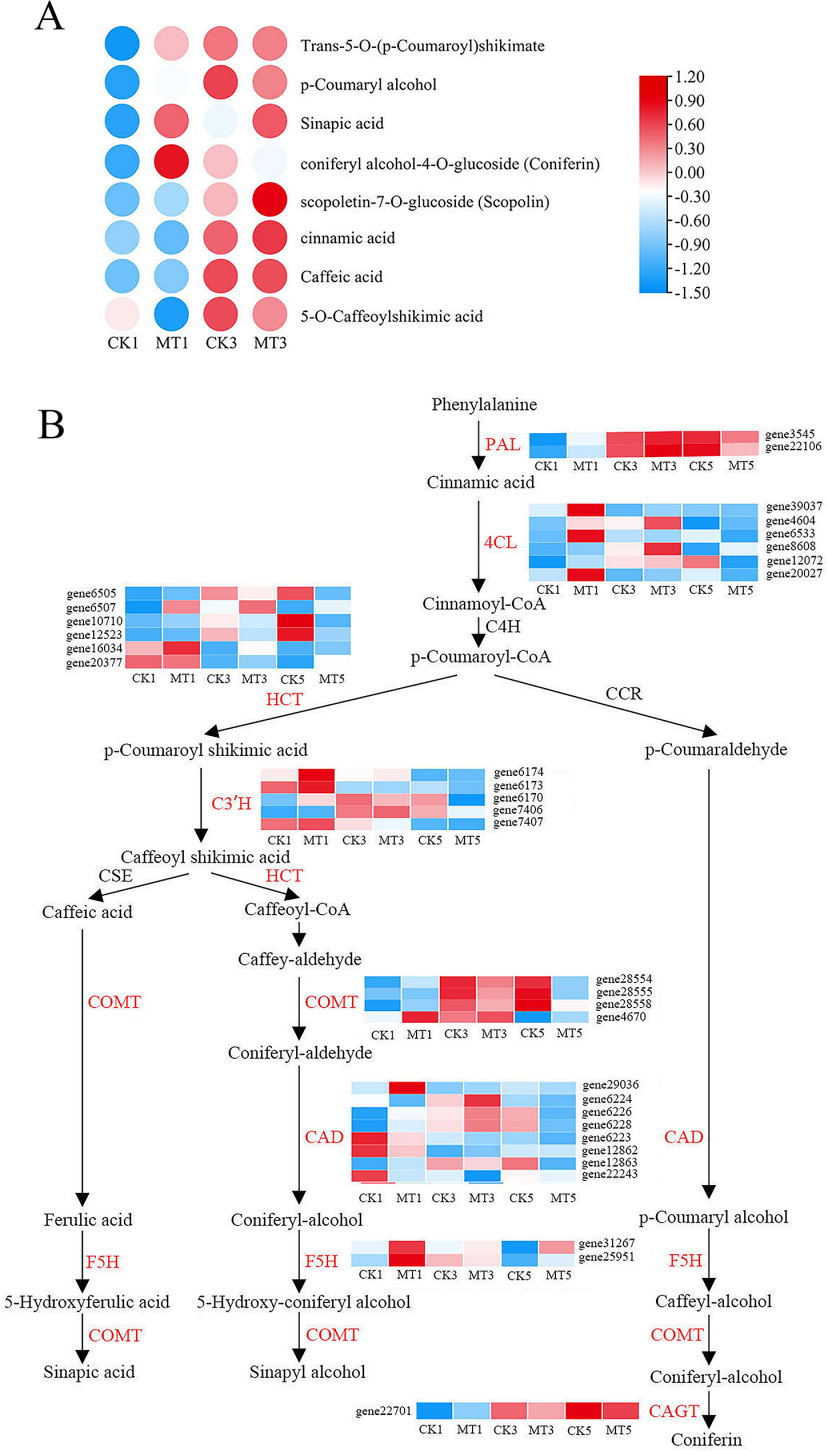
Melatonin treatment regulates the phenylpropanoid synthesis pathway. (**A**, **B**) Changes in the contents of phenylpropanoid biosynthesis pathway-related metabolites (**A**) and expression levels of phenylpropanoid biosynthesis genes (**B**) in ‘Whangkeumbae’ pear flesh after melatonin treatment

In pear fruits, attention has been paid to the significant increase in melatonin-induced differential metabolites in the phenylpropanoid, JA, and flavonoid biosynthesis pathways. Metabolomic analysis revealed that the contents of phenylpropanoid biosynthesis pathway-related metabolites, including trans-5-O-*p* coumarin shikimic acid, *p*-coumarin alcohol, erucic acid, and juniperin, significantly increased in pear fruits after melatonin treatment (Fig. 4A). Consistently, transcriptome analysis showed that key genes encoding phenylalanine ammonia lyase (*PAL*), coumarin 3-hydroxylase (*C3’H*), 4-coumarin CoA ligase (*4CL*), shikimate hydroxycinnamoyl transferase (*HCT*), caffeic acid methyltransferase (*COMT*), ferulic acid 5-hydroxylase (*F5H*), cinnamyl alcohol dehydrogenase (*CAD*), and coniferol alcohol glucosyltransferase (*CAGT*) were upregulated in the melatonin treated group (Fig. 4B).

**Fig. 5:**
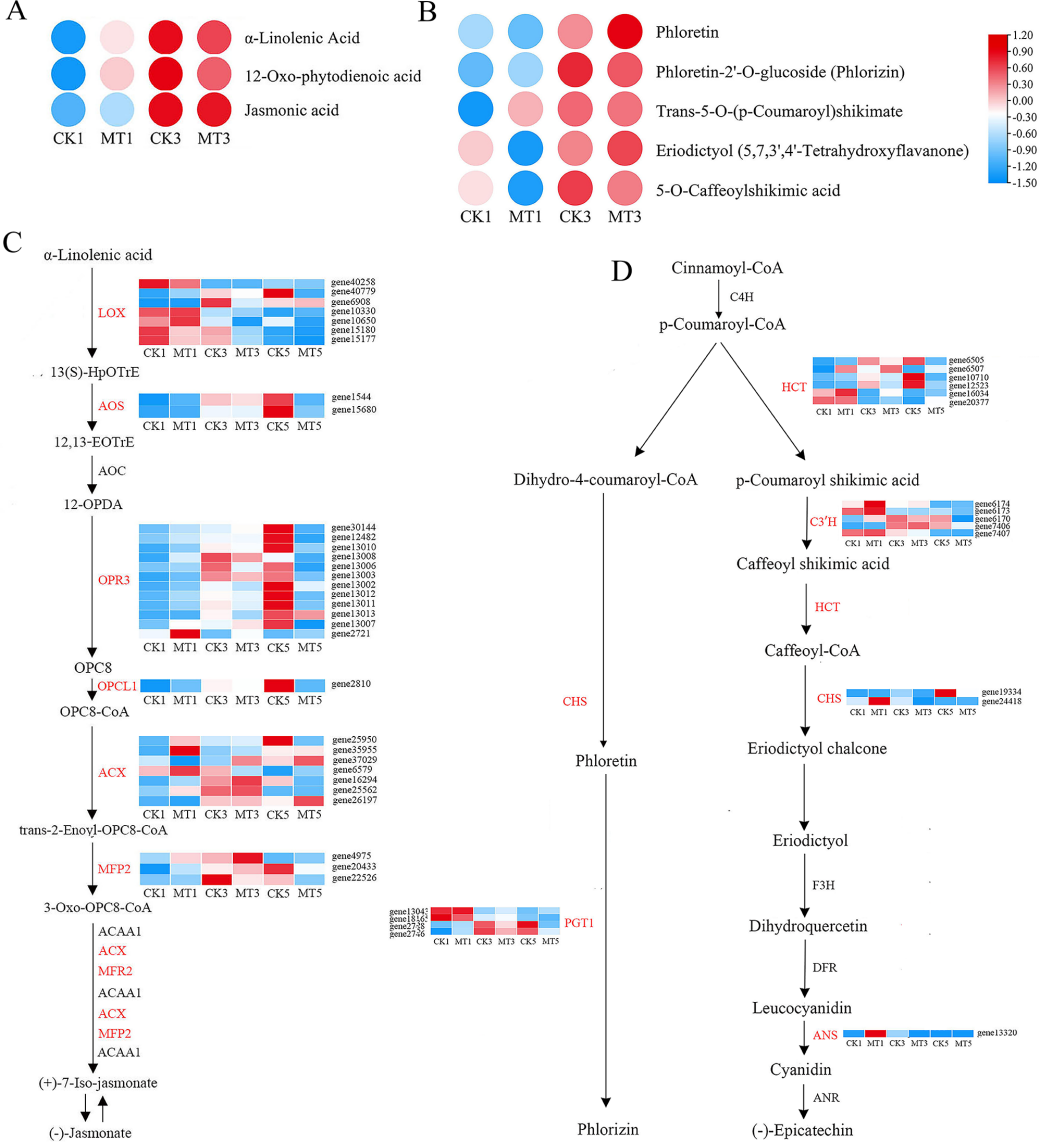
Melatonin promotes the biosynthesis of JA and phlorizin. (**A**, **B**) Contents of JA biosynthesis (**A**) and phlorizin biosynthesis (**B**) related metabolites. (**C**, **D**) Expression of JA biosynthesis (**C**) and phlorizin biosynthesis (**D**) related genes

The content of synthetic precursors of JA, α-linolenic acid and 12-oxo-phytodienoic acid, significantly increased on the first day after melatonin treatment. Consistently, the content of JA also significantly increased on day 1 (Fig. 5A). Genes encoding JA biosynthesis enzymes, such as lipoxygenase (*LOX*), allene oxide synthase 3-like (*AOS*), 12-oxophytodienoate reductase 3-like (*OPR3*), OPC-8:0 CoA ligase 1 (*OPCL1*), and acyl-CoA oxidase (*ACX*), were significantly upregulated on day 1. No significant difference was detected in gene expression between the treatment and control groups on day 3, but gene expression was higher in the control group than the treatment group on day 5 (Fig. 5C).

The contents of in the flavonoid biosynthesis pathway-related metabolites including trans-5-O-(*p*-Coumaroyl) shikimate, eriodictyol, phloretin, phlorizin, and 5-O-caffeoylshikimic acid significantly increased in pear fruits after melatonin treatment on day 1(Fig. 5B). Consistently, the expression of genes encoding flavonoid biosynthesis enzymes including *PpHCT*, *PpC3’H*, *PpCHS*, *PpANS*, and *PpPGT1* was also significantly upregulated after melatonin treatment (Fig. 5D).

### Effects of JA and phlorizin on the resistance of ‘Whangkeumbae’ pear fruit to *B. Dothidea*

**Fig. 6:**
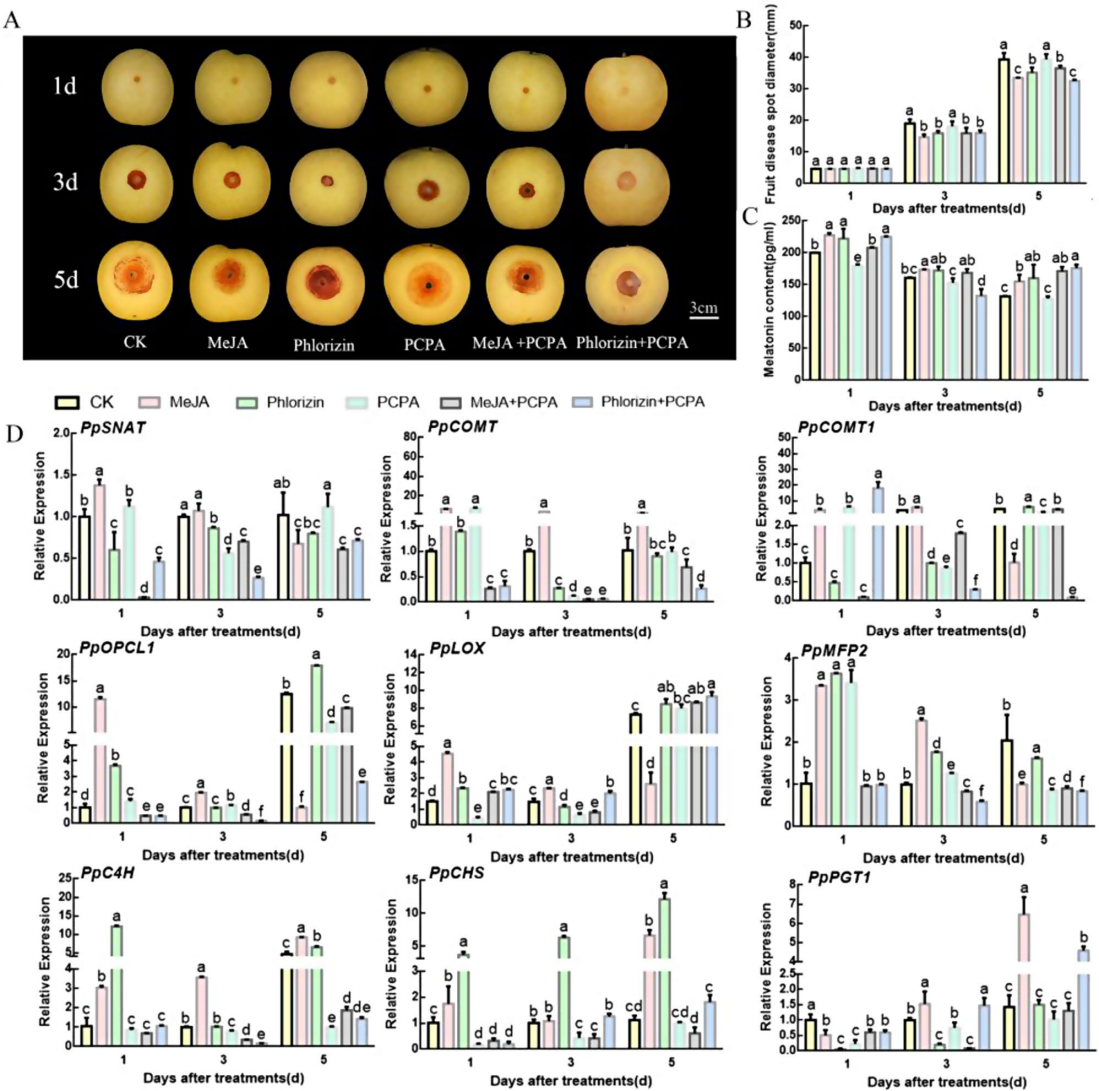
Phlorzin, JA, and MT synergistically promote resistance to *B. dothidea*. (**A**, **B**) Condition of *B. dothidea*-infected pear fruits after different treatments. (**C**) Content of melatonin in pear fruit. (**D**) Expression analysis of genes related to melatonin, JA, and phlorizin synthesis. Different letters indicate significant differences, according to one-way ANOVA Duncan’s multiple range test (*P* < 0.05)

After treatment with JA, the diameter of disease spots caused by *B. dothidea* infection decreased. JA significantly improved the resistance of pear fruit to *B. dothidea*, and phlorizin had the same effect. Notably, no significant difference was detected in the size of disease spots between control and PCPA-treated fruits; however, the content of melatonin was significantly reduced in the fruit. After co-treatment with PCPA and JA, the size of lesions was smaller than that of the control group. However, co-treatment with PCPA and phlorizin enhanced fruit resistance to *B. dothidea*. The fruit content of melatonin significantly decreased after PCPA treatment. In fruits treated with JA, phlorizin, and PCPA, the content of melatonin was high in comparison with control (Fig. 6A and C). Transcriptome data analysis showed that JA treatment significantly increased the expression of genes related to melatonin, JA, and anthocyanin biosynthesis such as serotonin N-acetyltransferase (*PpSNAT*), caffeic acid 3-O-methyltransferase (*PpCOMT*) and *PpCOMT1* in the first 3 days. *PpCOMT* was significantly upregulated in the first three days after treatment with phlorizin. PCPA treatment inhibited the expression of *PpSNAT*; however, *PpCOMT* was significantly upregulated on the first day and downregulated on the third day after PCPA treatment. Co-treatment with JA and PCPA significantly inhibited the expression of *PpSNAT* on the first day after treatment. The expression of phlorizin biosynthesis genes was also inhibited after JA and PCPA co-treatment. The combined treatment of phlorizin and PCPA significantly induced *PpCOMT1* on day 1 but significantly inhibited the synthesis of JA and phlorizin (Fig. 6D).

### Role of JA and phlorizin in improving pear resistance to *B. dothidea*

**Fig. 7:**
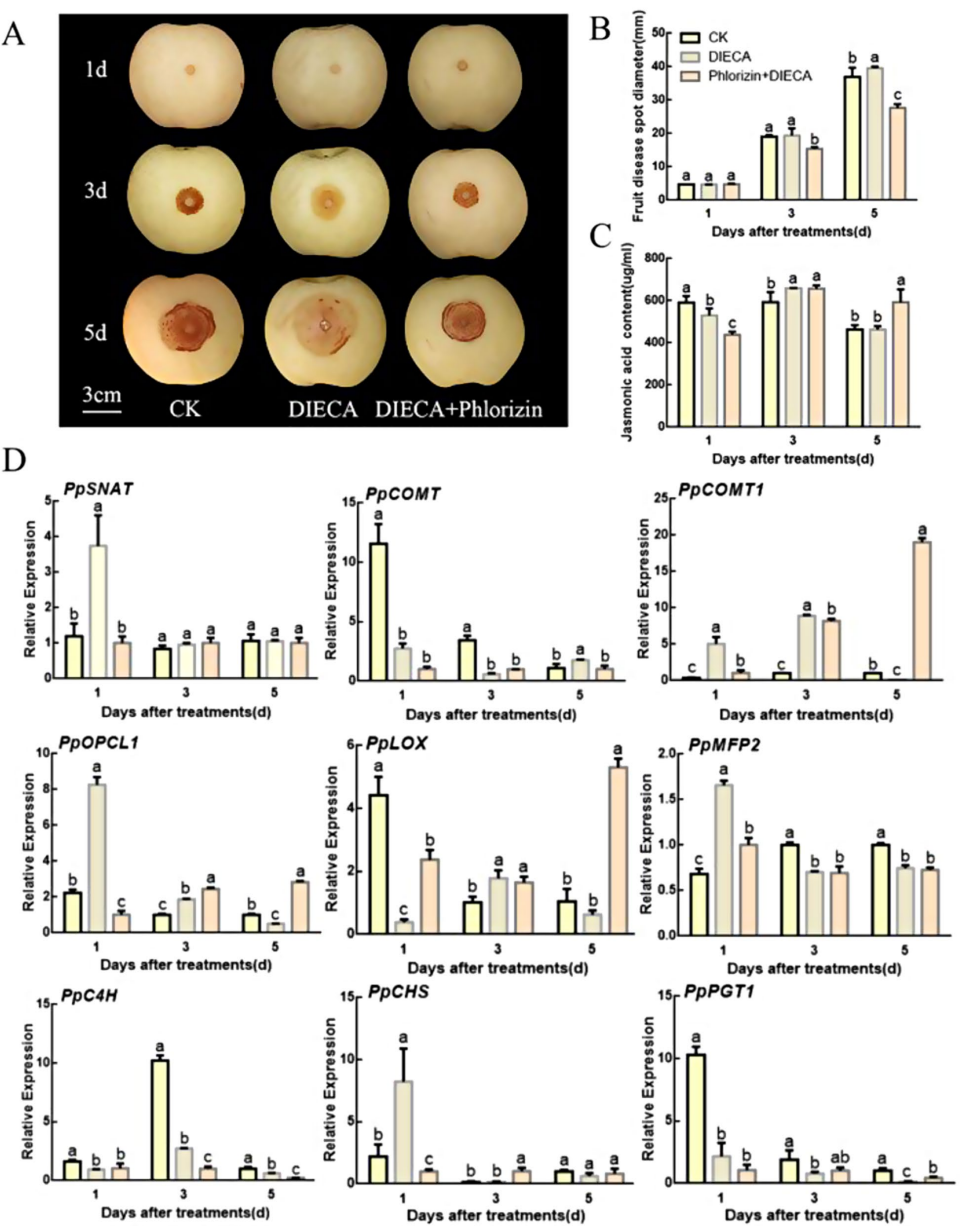
JA and phlorizin enhance the resistance of pear fruit to *B. dothidea*. (**A**, **B**) Changes in lesion size on *B. dothidea*-infected pear fruits after different treatments. (**C**) Content of JA in pear fruit. (**D**) Expression analysis of genes related to melatonin, JA, and phlorizin synthesis. Different letters indicate significant differences, according to one-way ANOVA Duncan’s multiple range test (*P* < 0.05)

Treatment of pear fruits with DIECA weakened the resistance to *B. dothidea* and significantly decreased the content of JA to levels lower than the control group. After joint treatment with DIECA and phlorizin, the diameter of fruit lesions decreased (Fig. 7A and B). DIECA inhibited the synthesis of JA, which was accompanied by a significant downregulation of *PpLOX* expression. After the addition of phlorizin, the content of JA remained significantly low on the first day but increased significantly on the fifth day after treatment. *PpOPCL1* and *PpLOX* were significantly upregulated on the fifth day, indicating that phlorizin stimulates JA biosynthesis (Fig. 7C and D).

## Discussion

Melatonin is widely present in bacteria, fungi, animals, and plants, and participates in regulating various biological processes [31–33]. In this study, exogenous melatonin application on pear fruits significantly increased their resistance to *B. dothidea*, which causes ring rot in plants. Therefore, we studied the effects of melatonin on *B. dothidea* pathogenesis and determined the role of melatonin in improving the resistance to *B. dothidea* in pear.

In this study, melatonin did not affect the growth of the pathogen during in vitro culture on PDA medium containing 50, 100, and 200 µmol/L concentrations of melatonin (Fig. 1). *B. dothidea* mycelia maintained an intact state and showed plump growth under different concentrations of melatonin, possibly because melatonin acts as a growth-promoting substance in *B. dothidea*. In recent studies, melatonin inhibited the normal growth of fungi, which may indicate that melatonin is selective in its response to fungal growth [34, 35].

Melatonin, as a novel plant growth regulator, has been extensively studied under biotic stress conditions. Currently, there are two main viewpoints regarding fungal stress: (1) melatonin enhances the antioxidant capacity of fruits, thereby increasing fungal stress resistance [36, 37], (2) melatonin increases fungus resistance in plants by increasing the content of defense related compounds, such as SA and JA, among others [38]. Therefore, we first verified the above viewpoint 1) by measuring the activity of antioxidant enzymes such as POD, CAT, and PPO. Melatonin treatment increased the activity of antioxidant enzymes in pear fruit (Fig. 2D-F). To gain a more comprehensive understanding of the impact of melatonin on the ring rot resistance of pear fruit, we analyzed the metabolic changes in melatonin-treated fruits infected with *B. dothidea*. On days 1 and 5, melatonin treatment significantly increased the content of JA in un-infected fruits (Fig. 5A). Additionally, the phenylpropanoid pathway, which is related to plant resistance, was also significantly induced on the first day(Fig. 5D). This indicates that melatonin can induce acquired resistance in plants. Furthermore, transcriptomic analysis revealed that genes related to JA and phenylpropanoid biosynthesis were upregulated in melatonin treated fruit on day 1 (Fig. 5C and D). However, on day 5, pathways related to disease resistance were also upregulated in the control group. This implies that although melatonin can trigger induced resistance in plants, it also weakens the sensitivity of plants to induced resistance after infection. These results demonstrate that melatonin can act as an acquired resistance stimulator in plants, and fruits should be treated with melatonin to prevent postharvest diseases.

Metabolomics analysis showed that melatonin enhanced the synthesis of JA, further proving that melatonin elicits induced systemic resistance in plants. Additionally, the content of phlorizin biosynthesis-related metabolites significantly increased. Phlorzin enhances pathogen resistance in plants. Oleszek et al. [39]. reported that components containing phloridzin in apple waste have the strongest antifungal activity. Recently, Mansoor et al. [40]. showed that the content of phloridzin significantly increased in apple plants infected with *Venturia inaequalis*, improving their defense ability. Therefore, we used the melatonin synthesis inhibitor PCPA and JA synthesis inhibitor DIECA to verify the relationship among melatonin, jasmonic acid, and phlorizin in improving the resistance of pear to *B. dothidea* [41–43]. After treatment with PCPA and JA, the melatonin content of pear fruit significantly increased, and the fruit regained some resistance to *B. dothidea* (Fig. 6A and B). Melatonin biosynthesis genes *PpSNAT*, *PpCOMT*, and *PpCOMT1* were significantly upregulated after JA treatment(Fig. 6D), indicating that JA enhances fruit resistance downstream of melatonin and can also promote melatonin synthesis, which implies a synergistic relationship between melatonin and JA. However, treatment with phlorizin and a combination of PCPA and phlorizin did not increase the melatonin content of pear fruit compared with the control group (Fig. 6C). Moreover, phlorizin treatment downregulated the expression of melatonin biosynthesis genes *PpSNAT*, *PpCOMT*, and *PpCOMT1* (Fig. 6D). Therefore, while it cannot be proven that phlorizin promotes melatonin synthesis, our results indicate that phlorizin functions downstream of melatonin. Further analysis of the relationship between JA and phlorizin using a co-treatment of DIECA and phlorizin revealed that the addition of JA biosynthesis inhibitors can still improve the resistance of pear fruit to *B. dothidea* (Fig. 7A and B); however, no clear evidence was available to support that phlorizin promotes JA biosynthesis, indicating that phlorizin functions downstream of JA.

## Conclusion

**Fig. 8:**
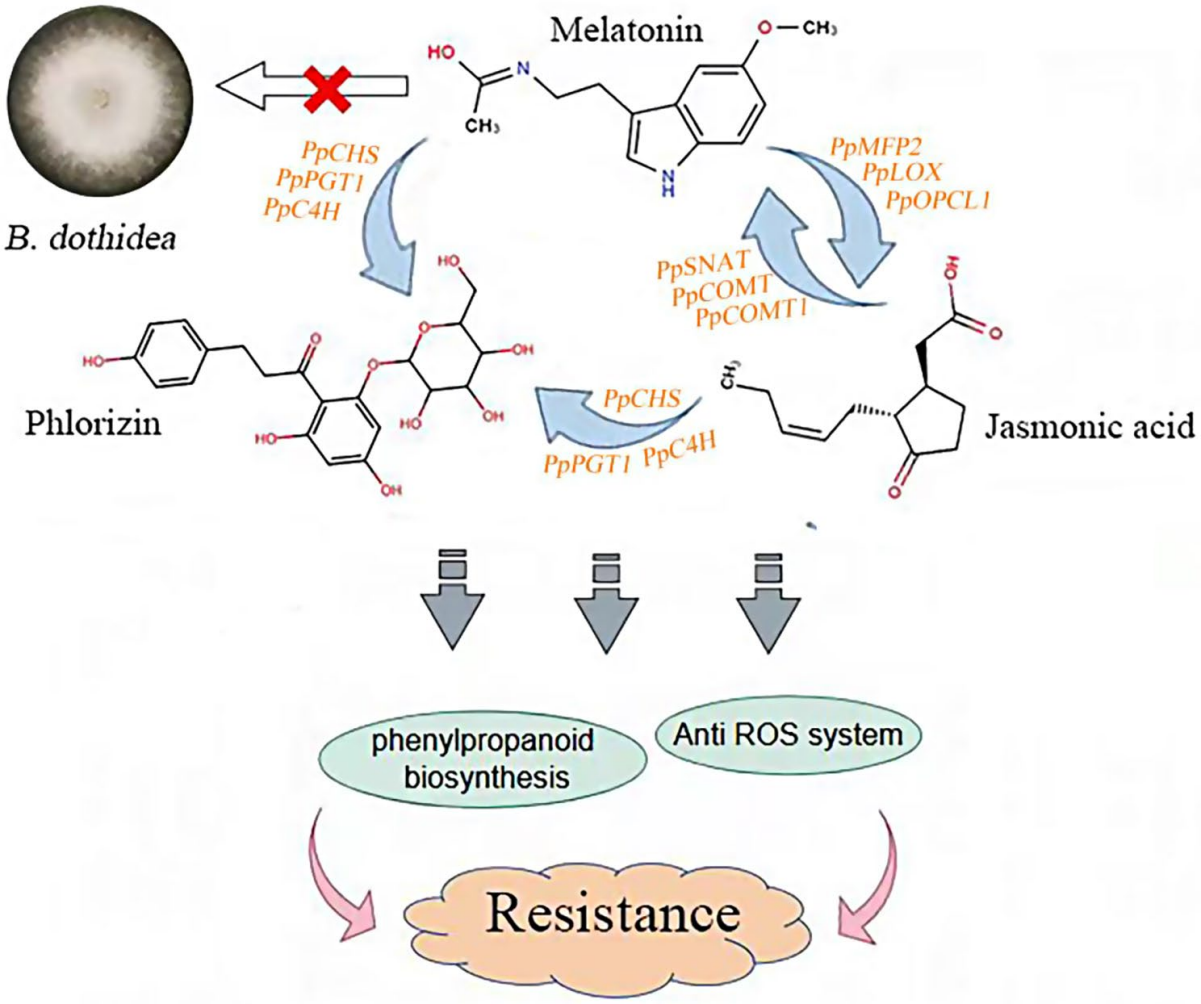
Proposed model for the mechanism of melatonin in improving the resistance of pear fruit to ring rot disease. In the model diagram of melatonin action, melatonin has no effect on the growth of *B. dothidea*. Melatonin can promote the synthesis of jasmonic acid and phlorizin in the fruit, all of which can improve the resistance of the fruit to ring rot disease. Jasmonic acid can feedback regulate the synthesis of melatonin, and also promote the synthesis of phlorizin. Ultimately, the resistance of the fruit is improved through antioxidant and phenylpropanoid metabolic pathways

In summary, this study systematically analyzed the function of melatonin in improving the resistance of pear fruit to ring rot. Our research confirms that melatonin has no effect on the growth of *B.dothidea in vitro*. However, melatonin can enhance the resistance of ‘Whangkeumbae’ pear to pear ring rot caused by *B.dothidea* by enhancing its antioxidant capacity and disease-resistant pathways such as phenylpropanoid biosynthesis pathway. It is worth noting that melatonin can activate induced resistance acquisition in a JA-dependent manner, and JA can also promote the synthesis of melatonin in fruits. Additionally, phlorizin, which acts downstream of JA and melatonin, increases fruit resistance to ring rot (Fig. 8).

### Electronic supplementary material

Below is the link to the electronic supplementary material.

Supplementary Material 1

## Data Availability

All data supporting the findings of this study are available within the paper and within its supplementary data published online. The data presented in the study are deposited in the National Center for Biotechnology Information (NCBI) BioProject database repository, accession number PRJNA1090158.
