# Supplementary material for: Melatonin enhances resistance to Botryosphaeria dothidea in pear by promoting jasmonic acid and phlorizin biosynthesis
Source: BMC Plant Biol. 2024 May 29;24:470. doi: 10.1186/s12870-024-05187-1 (PMC11134937; doi:10.1186/s12870-024-05187-1)
Supplement: Supplementary file 1 — Supplementary Material 1 [file 12870_2024_5187_MOESM1_ESM.docx]

Table S1 The primer sequences used for real-time qPCR

| Gene Name | Forward（5’-3’） | Reverse（5’-3’） |
| --- | --- | --- |
| *PbActin*  *PbSNAT*  *PbCOMT*  *PbCOMT1*  *PbOPCL1*  *PbLOX*  *PbMFP2*  *PbC4H*  *PbCHS*  *PbPGT1* | ACAGTGTCTGGATTGGAGGGTC  GCTGCTGTGGATGGAGTACA  TTGGTATGCTCCCATCTGCAT  TGGTGCTGGTTTTAGTGGCA  GATTCGCTGGAGGAGAGTCG  AACGCACTTGCACGACAAAG  CCCAAGAAAAGTTTGAGAAGACGA  CCTTCTGGGTCTCTTCGTCG  GAGGAAGTTCGCAAGGCTCA  AGTTTCAAAGACCTCCGCGA | CATTTGGAGAACTCAGAAGCACT  ACCACGTCCCATATGATCGC  TGGGGGAAAATCCAGGTACG  TTTGAAAGCACTCTTGTCTACTTGT  CCGTCTTATTCGCCGCTAGT  ATGCTGCAGAGCTTACCTCC  AACCTTCTCAATAACGGCCTCT  GACCTGGAGCCAGTTTCCAA  CGGGGTATGTAGCTTGGTCC GTCCGAATAAGCAGGGTCGT |

Figure.S1


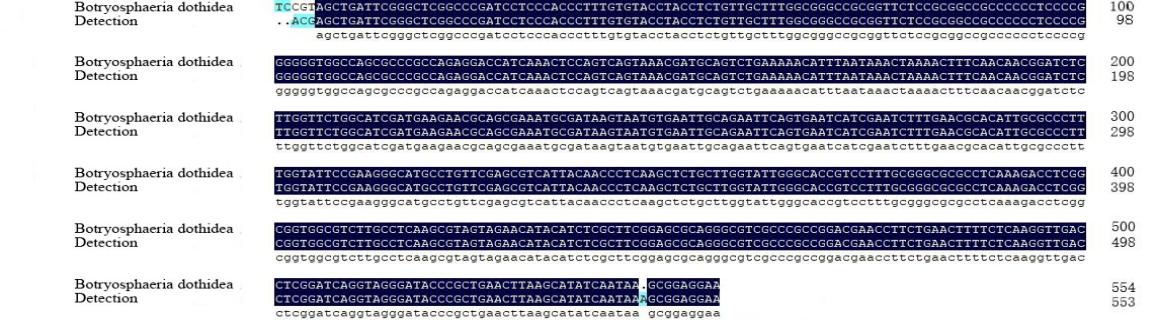


Figure.S1 The Strains used for ITS sequence alignment

Figure.S2


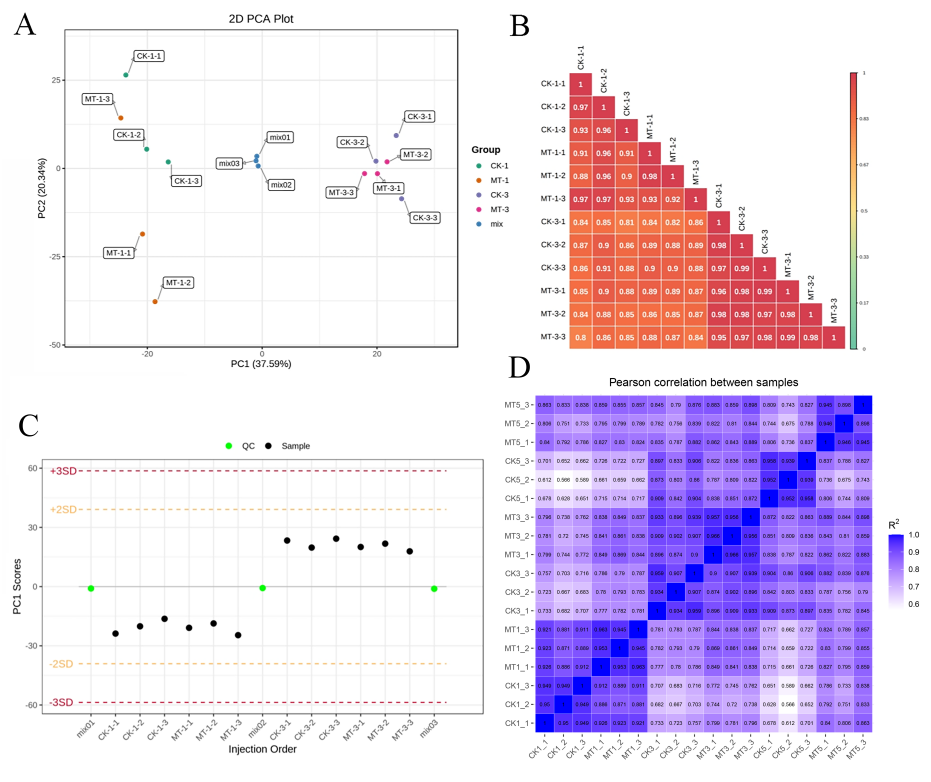


Figure.S2 PCA score chart of mass spectrometry data between each group of samples and quality control samples (A) in metabolomics, PC1 control chart of overall samples (C), and correlation chart between samples (B) . Correlation map between samples in transcriptomics (D).
